# Transcriptome assembly from long-read RNA-seq alignments with StringTie2

**DOI:** 10.1186/s13059-019-1910-1

**Published:** 2019-12-16

**Authors:** Sam Kovaka, Aleksey V. Zimin, Geo M. Pertea, Roham Razaghi, Steven L. Salzberg, Mihaela Pertea

**Affiliations:** 1 Department of Computer Science, Johns Hopkins University, Baltimore, MD 21218 USA; 2 Center for Computational Biology, Whiting School of Engineering, Johns Hopkins University, Baltimore, MD 21205 USA; 3 Department of Biomedical Engineering, Johns Hopkins University, Baltimore, MD 21218 USA; 4 Department of Biostatistics, Bloomberg School of Public Health, Johns Hopkins University, Baltimore, MD 21205 USA

**Keywords:** Transcriptome assembly, RNA-seq, Long-read sequencing, Gene expression

## Abstract

RNA sequencing using the latest single-molecule sequencing instruments produces reads that are thousands of nucleotides long. The ability to assemble these long reads can greatly improve the sensitivity of long-read analyses. Here we present StringTie2, a reference-guided transcriptome assembler that works with both short and long reads. StringTie2 includes new methods to handle the high error rate of long reads and offers the ability to work with full-length super-reads assembled from short reads, which further improves the quality of short-read assemblies. StringTie2 is more accurate and faster and uses less memory than all comparable short-read and long-read analysis tools.

## Background

Measuring the abundances of transcripts in an RNA-sequencing (RNA-seq) dataset is a powerful way to understand the workings of a cell. Simply aligning reads to a reference genome can provide rough estimates of the average expression of genes and hint at differential use of splice sites [1], but to create an accurate picture of gene activity, one must assemble collections of reads into transcripts. Alternative splicing is very common in eukaryotes, with an estimated 90% of human multi-exon protein-coding genes and 30% of non-coding RNA (ncRNA) genes having multiple isoforms [2, 3]. While the number of annotated human protein-coding genes has remained more or less constant over the last decade, the number of ncRNA genes and protein-coding isoforms has continued to increase [4].

Second-generation sequencers, such as those from Illumina, can produce hundreds of millions of short (~ 100 bp) RNA-seq reads. Reads of this length usually span no more than two exons, except in cases of very small exons. By assembling the short reads, we can reconstruct full-length transcripts and identify novel genes and gene isoforms. There are two main approaches to transcriptome assembly: de novo and reference-guided. De novo transcriptome assemblers such as Trinity [5] and Oases [6] find overlaps between reads and attempt to chain them together into full transcripts, without aligning the reads to a genome. This task is complicated by the presence of paralogous genes and transcripts with many isoforms that largely overlap one another, and as a result, this approach produces highly fragmented and error-prone transcriptomes. Reference-guided assemblers such as Cufflinks [7], Bayesembler [8], StringTie [9], TransComb [10], and Scallop [11] take advantage of an existing genome to which the RNA-seq reads are first aligned using a spliced aligner such as HISAT [12] or STAR [13]. These assemblers can then build splice graphs (or other data structures) based on the alignments and then use those graphs to construct individual transcripts. Some reference-guided assemblers can also use the exon-intron annotation of known transcripts as an optional guide, allowing them to favor known genes where possible. A recent study [14] found that StringTie outperforms both Cufflinks and Bayesembler, by assembling more transcripts correctly and at a higher precision, while the original Scallop study [11] showed that on some datasets, Scallop can achieve higher sensitivity and precision than StringTie (version 1.3) and TransComb.

StringTie and other transcriptome assemblers estimate transcript abundance based on the number of aligned reads assigned to each transcript. More recently, alternative methods such as Sailfish [15], Salmon [16], and Kallisto [17] demonstrated that one can estimate abundances by assigning reads to known transcripts based on exact *k*-mer matching, which produces dramatic gains in speed by dropping the requirement for precise base-level read alignment. However, these alignment-free methods are not able to detect novel genes or isoforms, and they show poorer performance in quantifying low-abundance and small RNAs compared to alignment-based pipelines [18].

The original release of StringTie proposed a method to use a limited version of de novo transcriptome assembly via the construction of super-reads, which were originally developed for whole-genome assembly [19]. Conceptually, super-reads are constructed by extending each end of a short read as long as there is a unique extension based on a *k*-mer lookup table. This creates a collection of synthetic long reads with the low error rate of short reads. Because they are longer, they are more likely to align uniquely to the genome, which in turn might simplify the splice graph of a gene. Super-reads were used in a limited capacity in StringTie 1.0 (henceforth StringTie1), only filling in the gap between paired-end reads. In that limited implementation, a super-read was used to replace a pair of reads, allowing it to be treated like a single, unpaired read. One difficulty in using super-reads is that the algorithm used to create them for genome assembly includes an error correction step, which in the context of RNA-seq assembly can over-write *k*-mers from low-abundance transcripts. Another complication is that a full super-read may contain many short reads, and thus, it cannot be counted as a single read during the quantification step. We have therefore developed an expectation-maximization (EM) algorithm to distribute read coverage between super-reads.

While second-generation sequencers produce very large numbers of reads, their read lengths are typically quite short, in the range of 75–125 bp for most RNA-seq experiments. These short reads often align to more than one location, and we designate such reads as “multi-mapping.” Short reads also suffer the limitation that they rarely span more than two exons, making the splice graph difficult and sometimes impossible to traverse accurately for genes with multiple exons and many diverse isoforms, no matter how deeply they are sequenced. These issues can be alleviated by third-generation sequencing technologies such as those from Pacific Biosciences (PacBio) and Oxford Nanopore Technologies (ONT). These long-read technologies, which can produce read lengths in excess of 10,000 bp, have dramatically improved whole-genome assemblies [20], and when used for RNA-seq experiments, they offer the potential for large gains in the accuracy of isoform identification and discovery [21–23]. While some reads produced by third-generation sequencers cover the full length of RNA transcripts, many will inevitably capture only partial transcripts. This happens for a variety of reasons, e.g., (1) RNA degrades quickly and may be shorter than full length by the time it is captured for sequencing; (2) long molecules can break during library preparation; or (3) in cDNA sequencing, the reverse transcription step may fail to capture the full RNA molecule. Thus, computational tools that only consider reads which fully cover a transcript will be forced to discard many reads, possibly causing a substantial reduction in sensitivity. To date, though, long reads have not been widely adopted for transcriptome assembly, in part because they have a much higher error rate (typically 8–10% or higher), making alignment difficult [24, 25], and also because long-read sequencers have much lower throughput, which makes accurate quantification of all but the highest-expressed genes impossible.

Various tools have recently been developed to correct errors and/or extract full-length transcripts from genome alignments of long RNA-seq reads. Tools that process full-length transcripts from PacBio Iso-Seq reads, including ToFU [26], TAPIS [27], and SQANTI [28], cannot assemble reads that only partially cover transcripts into full-length transcripts, nor can they be applied to ONT reads due to their reliance on identifying 5′ and 3′ ends based on PacBio-specific adapters. TranscriptClean [29] corrects mismatches, indels, and non-canonical splice-sites in long-read alignments, but does not attempt to identify full-length transcripts. FLAIR [30] corrects splice-sites based on known, user-provided annotation and outputs transcripts from the annotation that are fully covered by “high-confidence” reads. As an alternative to these approaches, which depend on known transcripts, one can assemble long-read fragments using the same methods used for short-read transcriptome assembly. In addition to finding novel transcripts, the assembly approach can more readily handle fragments that match multiple isoforms, and it can correct alignment errors by forming a consensus from multiple reads. Traphlor [31] is the only previously described system designed to assemble high-error long reads, although we show it performs relatively poorly on both simulated and real data.

Here we present StringTie2, a major new release of the StringTie transcript assembler, which is capable of assembling both short and long reads, as well as full-length super-reads. Our results on 33 Illumina RNA-seq datasets demonstrate that StringTie2 is more accurate than Scallop, the next-best performing transcriptome assembler of those currently available. The use of super-reads also consistently improves both the sensitivity and precision of StringTie2 assemblies. When applied to long reads, StringTie2 assembles the reads substantially more accurately, faster, and using less memory than FLAIR, the next-best performing tool for long-read analysis. As opposed to FLAIR, StringTie2 can also identify novel transcripts from the long-read data, even when no reference annotation is provided.

## Results

### Transcriptome assembly of short RNA-seq reads

**Fig. 1:**
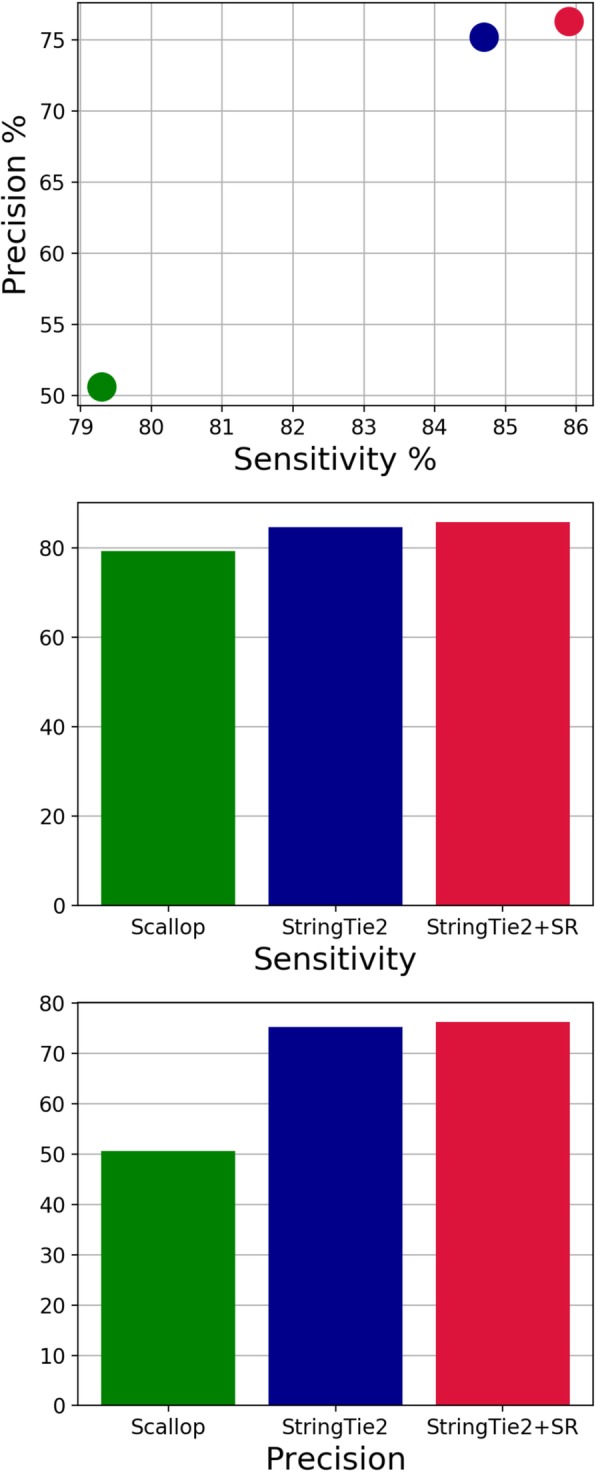
Sensitivity and precision of Scallop, StringTie2, and StringTie2 with super-reads (StringTie2 + SR) on simulated human short-read data, containing 150 million 75-bp paired-end reads. Only those transcripts that were completely covered by input reads were used in computing accuracy

We first used simulated human data to compare the sensitivity and precision of StringTie2, with and without super-reads, to that of Scallop (Fig. 1), one of the most recent transcriptome assemblers for short RNA-seq data, which was shown on some data to yield an improvement in assembly accuracy over StringTie1 [11].

We define sensitivity as the percent of expressed transcripts that match a transcript predicted (or output) by each tool and precision (equivalently called positive predictive value) as the percent of predicted transcripts that match an expressed transcript. We tuned the default parameters of StringTie2 to have approximately the same precision as StringTie1 (version 1.3) on this simulated data. StringTie2, with default parameters, is both more sensitive and more precise than Scallop on this data, and the use of super-reads increases both the sensitivity and precision of StringTie2 compared to using short-read alignments alone. Note that on this data, StringTie2 is also more sensitive and more precise than StringTie1 (Additional file 1: Figure S1). We also computed the Spearman correlation coefficients of the expression levels predicted by each tool compared to the true expression levels on simulated data (Table 1). StringTie2’s estimates of expression levels have a higher correlation than Scallop over all predicted and expressed transcripts, and the use of super-reads improves this correlation further.

**Table 1:** Spearman correlation coefficients for the performance of Scallop, StringTie2, and StringTie2 with super-reads on simulated short-read data. “Spearman predicted” only includes transcripts that each tool assembled. For non-assembled transcripts in “Spearman all,” the predicted expression was set to zero

	Spearman all	Spearman predicted
Scallop	0.726	0.828
StringTie2	0.781	0.925
StringTie2+SR	0.788	0.930

We next evaluated performance on real short-read data, which is considerably more complex than simulated data. For the real data, we cannot know with certainty which transcripts were expressed in each dataset, nor can we know their precise expression levels. However, it is generally safe to assume that an assembler is more sensitive if it assembles more transcripts matching known annotations (i.e., transcripts from a published database of known genes), and that it is more precise if the known transcripts represent a higher proportion of all the transcripts that are output by the assembler. Therefore, for each sample, we calculate sensitivity and precision by considering the number of assembled transcripts that match annotations as correct ones (i.e., true positives), and counting all additional transcripts as incorrect (also see the “Methods” section).

**Fig. 2:**
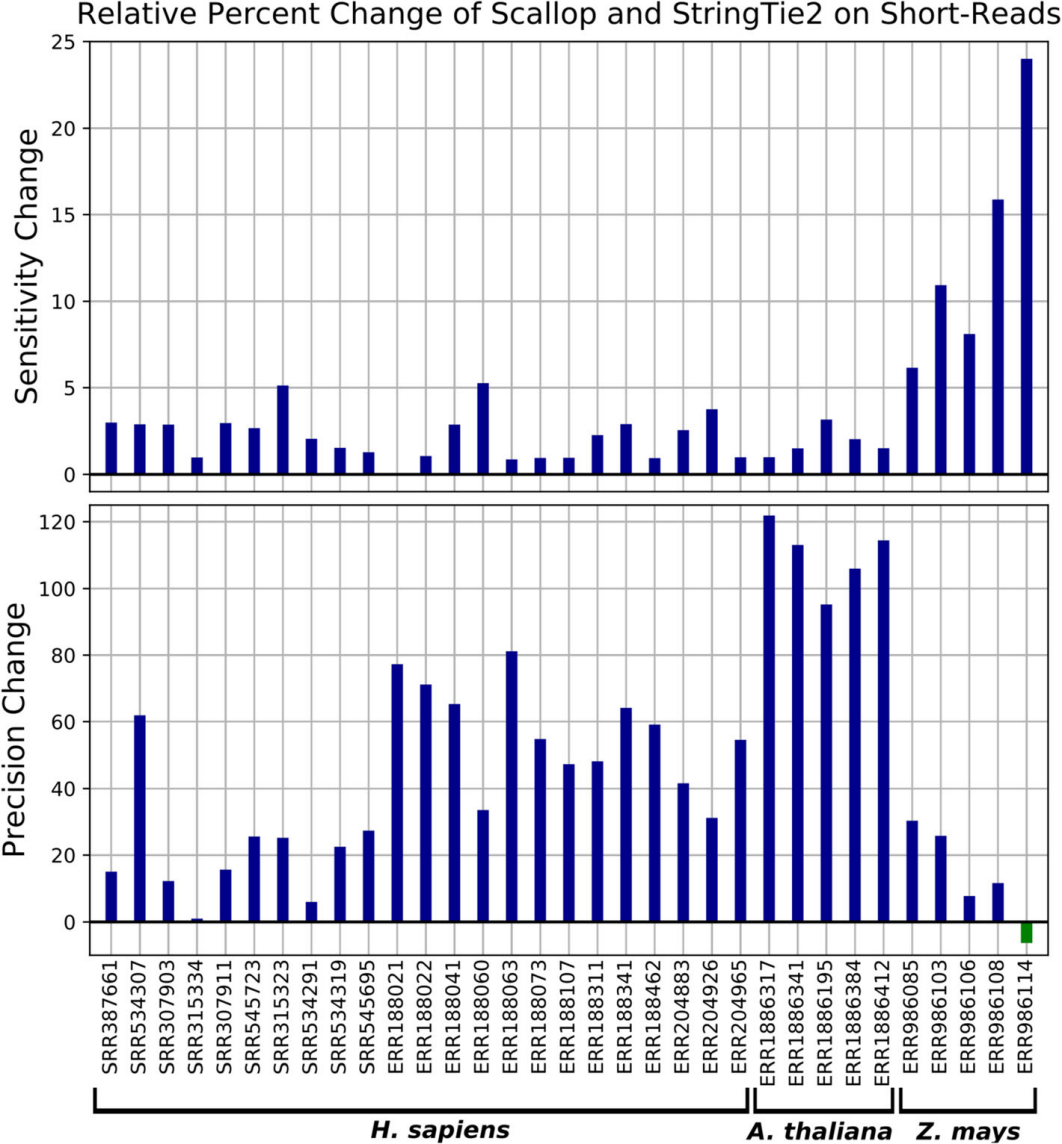
Relative change in sensitivity and precision of StringTie2 vs. Scallop on 23 real short-read RNA-seq datasets from human, five datasets from *Arabidopsis thaliana*, and five datasets from *Zea mays*. Positive values indicate that StringTie2 had an increase in sensitivity or precision, while negative values (for one dataset only) indicate lower precision

We ran StringTie2 and Scallop on 23 short-read datasets from human, five from *Arabidopsis thaliana*, and five from *Zea mays* (see the “Methods” section). Note that our datasets included all ten human samples that were used by Shao and Kingsford to show that Scallop is more sensitive than StringTie while maintaining the same level of precision [11]. StringTie2 obtained both better sensitivity and precision than Scallop on all 23 human datasets tested and 9/10 plant datasets (Fig. 2 and Additional file 2: Table S1). On average, StringTie2 obtained a relative increase over Scallop of 3.9% in sensitivity and 47.3% in precision. On the one *Zea mays* dataset (ERR986114) where Scallop had slightly higher precision than Stringtie2 (17.4% vs 16.3%), StringTie2 obtained a 24% relative increase in sensitivity (Fig. 2). Close inspection of the read alignments from this sample in IGV [32] revealed that there were many gaps in coverage within individual transcripts, possibly due to the highly repetitive nature of the *Z. mays* genome. Examining finer-grained statistics revealed that the intron-level precision of StringTie2 was higher than Scallop on this dataset (Additional file 2: Table S2), suggesting that the reduced transcript-level precision could be due to split transcripts caused by drops in coverage, which would result in fewer full-length transcript matches while leaving most introns intact. In fact, adjusting corresponding parameters in StringTie2 and Scallop to allow for larger gaps in coverage (see the “Methods” section) resulted in higher sensitivity and precision for StringTie2 on all *Zea mays* datasets (Additional file 2: Table S3).

StringTie2 is not only more accurate than Scallop, but also more time and memory efficient. Averaging over all real short-read datasets, StringTie2 ran 1.8 times faster than Scallop and used 17 times less memory (Additional file 2: Table S4).

**Fig. 3:**
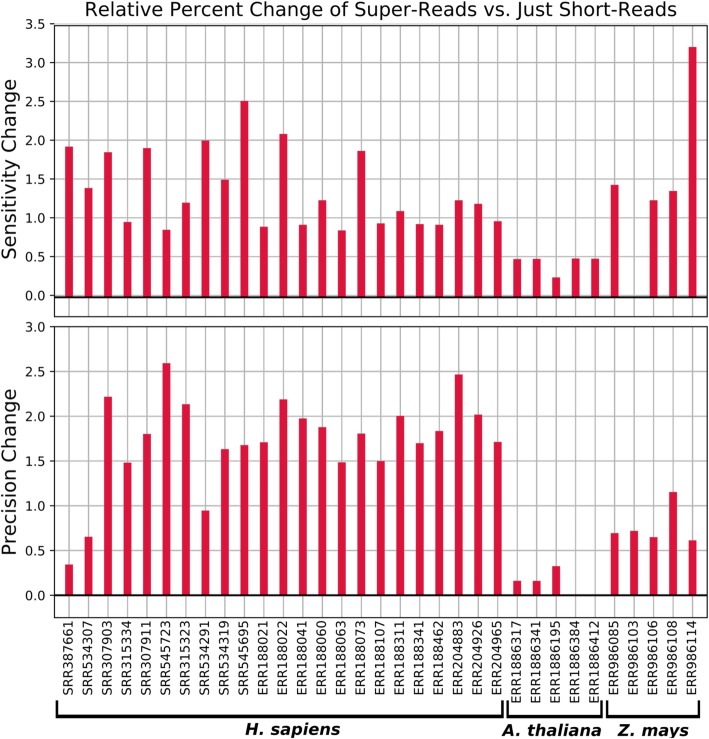
Relative change in percent sensitivity and precision when using super-reads on 23 real short-read RNA-seq datasets from human, five datasets from *Arabidopsis thaliana*, and five datasets from *Zea mays*. Positive values indicate that the use of super-reads increased sensitivity or precision

The use of super-reads increased both the sensitivity and precision of StringTie2 on all human datasets and all but three plant datasets (Fig. 3). Among those three, StringTie2 had an increase in precision but no change in sensitivity on one *Z. mays* dataset, and an increase in sensitivity but no change in precision on two *Arabidopsis* datasets.

### Transcriptome assembly of third-generation RNA-seq long reads

**Fig. 4:**
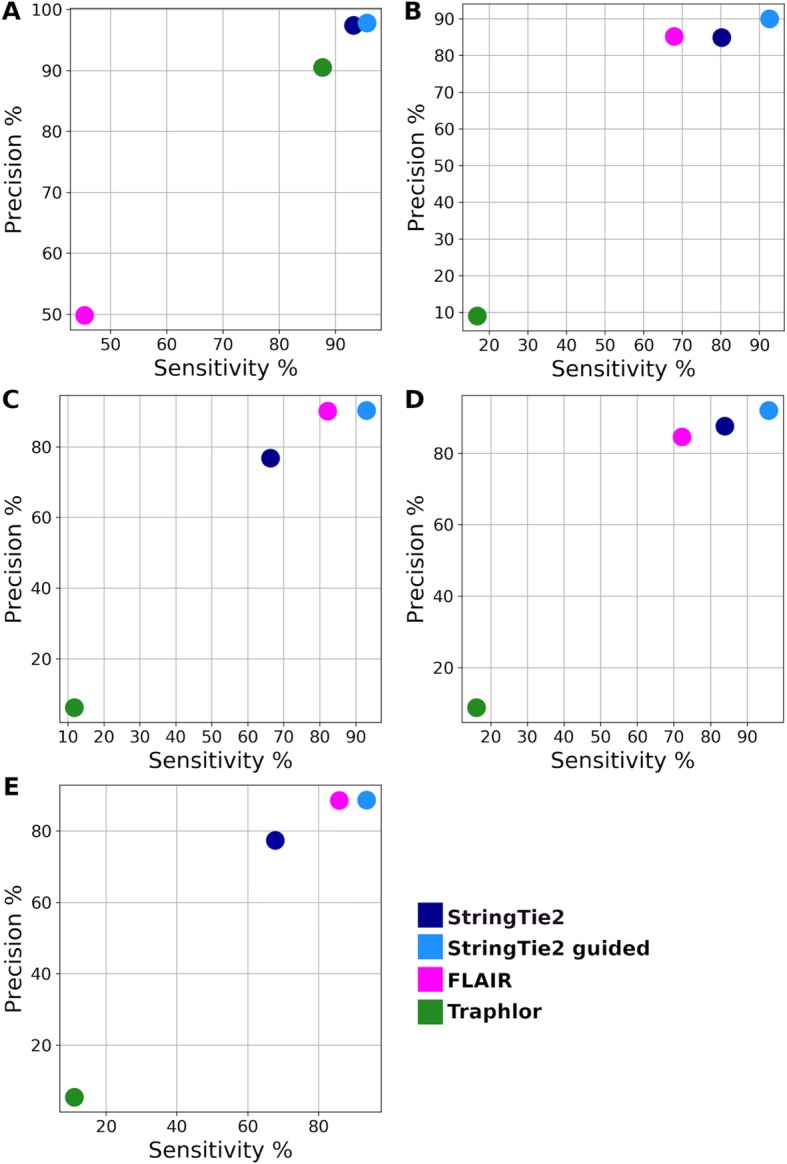
Sensitivity and precision of StringTie2 (with and without guide annotation), FLAIR, and Traphlor on long read simulated data from **a** PacBio *Saccharomyces cerevisiae*, **b** PacBio *Drosophila melanogaster*, **c** PacBio *Homo sapiens*, **d** ONT *D. melanogaster*, and **e** ONT *H. sapiens*

We next compared StringTie2’s performance on long-reads with that of FLAIR and Traphlor, the only other systems that can process both PacBio and ONT long-read RNA sequencing data. Because we cannot know the true transcripts that are present in real RNA-seq data sets, we first used simulated data to assess the accuracy of all tested tools. We obtained five simulated datasets generated by [33], who used the DNA simulator PBSIM [34] tuned to mimic the characteristics of either PacBio or ONT RNA-seq data. These datasets consist of a *Saccharomyces cerevisiae* PacBio run, two *Drosophila melanogaster* runs (one PacBio, one ONT), and two human chromosome 19 runs (one PacBio, one ONT). We ran StringTie2, FLAIR, and Traphlor on these simulated datasets and computed sensitivity and precision as before. FLAIR requires gene annotation as a guide to alignment, so we also ran StringTie2 with the same guide annotation in order to make a direct comparison. Results are shown in Fig. 4.

Traphlor had lower sensitivity and precision than StringTie2 and FLAIR on all datasets except for the *S. cerevisiae* PacBio data (Fig. 4a). Note that only 4.4% of the simulated transcripts in the *S. cerevisiae* PacBio dataset contain multiple exons, a much smaller proportion than the other datasets, which may explain why Traphlor performed relatively well on this data while performing poorly on the human and Drosophila data. StringTie2 with annotation as a guide outperformed FLAIR on all datasets, and in some cases, StringTie2 *without* guide annotation performed equally well. Because this was simulated data, the guide annotation included all transcripts that were present in the sample, even if not all of them were expressed. Real datasets are likely to contain unannotated transcripts and may lack many known, annotated genes entirely.

**Fig. 5:**
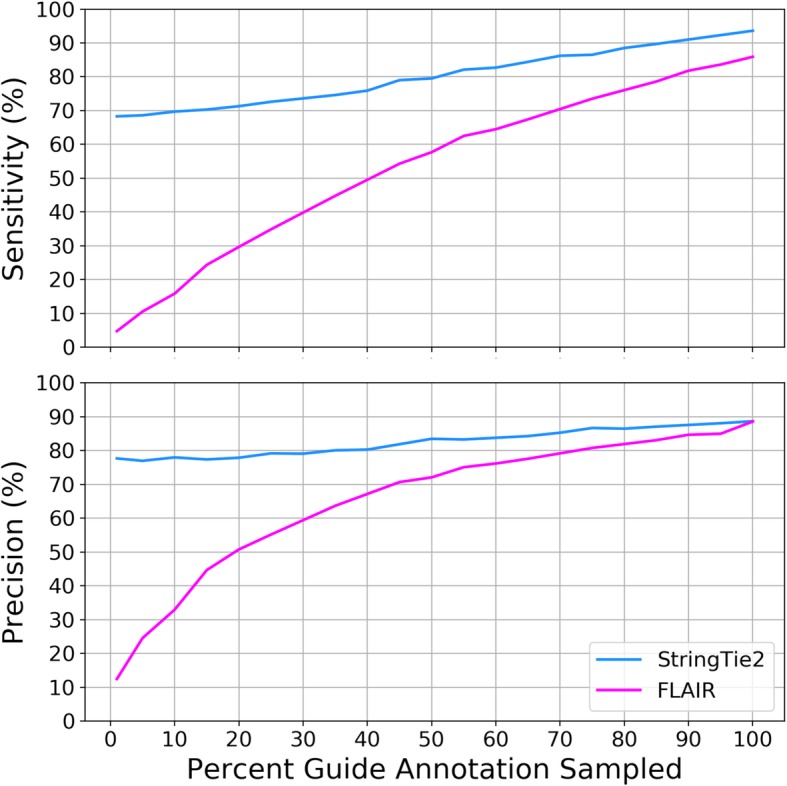
Sensitivity and precision of StringTie2 and FLAIR running on simulated ONT data from human chromosome 19, using random samples of different proportions of the human chromosome 19 annotation as a guide. e.g., 50% guide annotation on the *x*-axis shows results when both programs were provided 50% of the annotated genes as input

To demonstrate the performance of each tool when transcripts are missing from the guide annotation, we ran StringTie2 and FLAIR on the human chromosome 19 ONT data using random samples of the chromosome 19 annotation, which we varied to contain from 1 to 100% of the transcripts. Results are shown in Fig. 5. The sensitivity and precision of FLAIR decreases rapidly as the amount of annotation is reduced, e.g., when only 20% of the annotation is provided, FLAIR’s sensitivity and precision dropped to 30% and 50% respectively. In contrast, with that same amount of annotation, StringTie2’s results were far better, 74% and 80%. This result demonstrates FLAIR’s strong reliance on the guide annotation and StringTie2’s contrasting ability to assemble transcripts that are not present in the annotation.

**Fig. 6:**
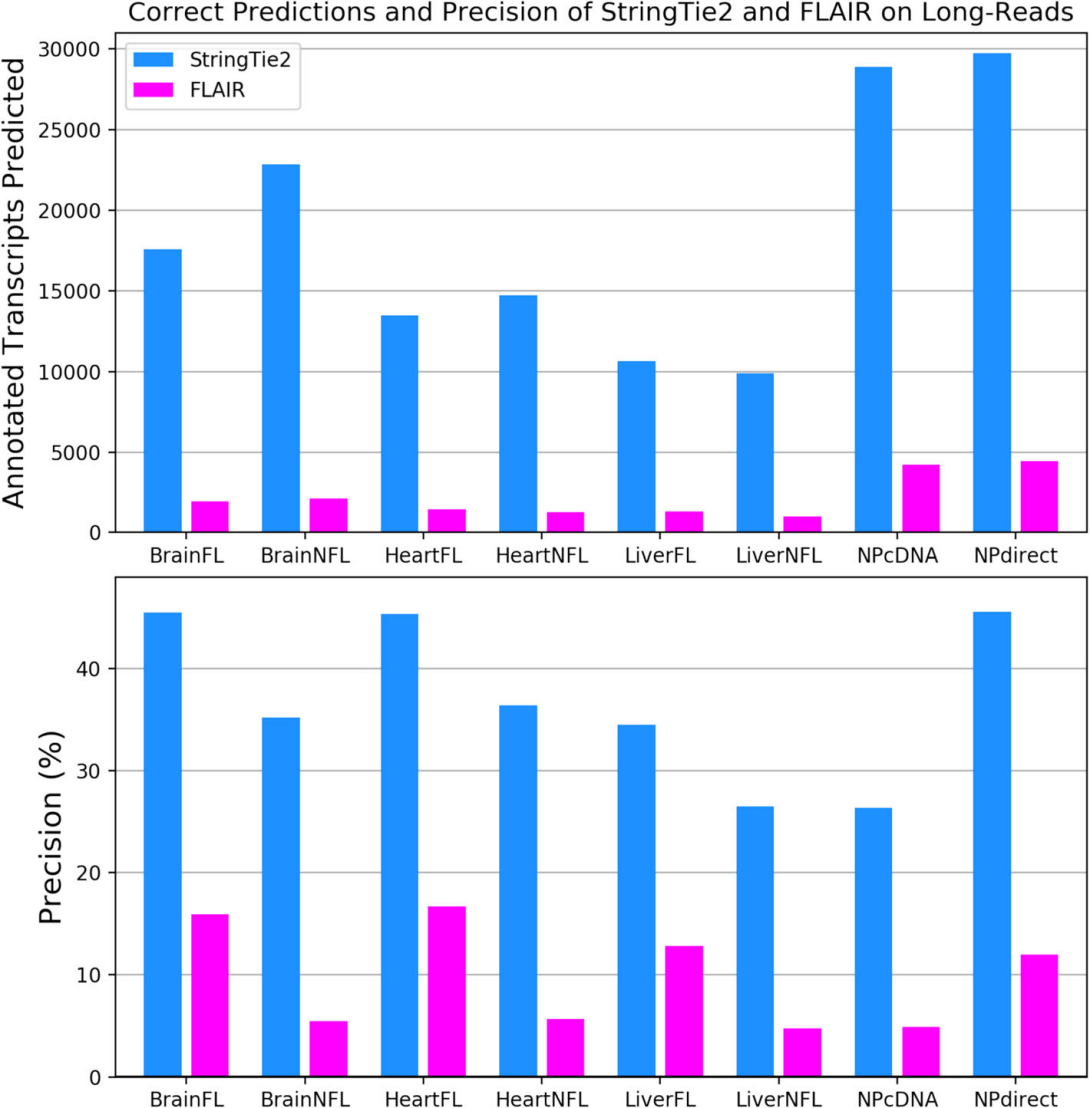
Number of correctly assembled transcripts and precision of StringTie2 (blue) and FLAIR (magenta) on real PacBio (FL = full length, NFL = not full length) and ONT (NP direct RNA and cDNA) human data. For both plots, any transcripts assembled by either tool were considered correct if it exactly matched all introns from a known, annotated transcript. In the lower plot, precision is defined as the percentage of all transcripts assembled by a program that match known annotation. Any additional transcripts will reduce precision if they do not match known transcripts, even if they are correct

We next ran StringTie2, FLAIR, and Traphlor on eight real human long-read datasets: three PacBio datasets enriched for full-length transcripts (PacBioFL), three PacBio datasets containing transcript fragments (PacBioNFL), one Nanopore cDNA dataset (NPcDNA), and one Nanopore direct RNA-seq dataset (NPDirect). Traphlor failed to produce any transcripts on the NPcDNA dataset and had drastically worse precision and sensitivity compared to StringTie2 on all other datasets (Additional file 2: Table S5). Averaging across all datasets on which Traphlor was able to run, StringTie2 correctly assembled 9564 transcripts, 2.6 times more than Traphlor’s 3708 correct assemblies. Compared to FLAIR, StringTie2 with guide annotation correctly identified 16,000 more transcripts on average, with precision that ranged from about three to six times higher (Fig. 6, Additional file 2: Table S5). FLAIR performed the best on the Nanopore direct RNA-seq dataset, where it correctly identified 4442 transcripts matching the annotation. By comparison, StringTie2 correctly assembled 29,744 transcripts, 6.7 times more than FLAIR. Even without using guide annotation, StringTie2 substantially outperformed FLAIR on all of the real datasets (Additional file 2: Table S5). Finally, StringTie2 with annotation runs 68 times faster than FLAIR and uses 9 times less memory, averaged over all real long-read datasets. Without annotation, StringTie2 is 93 times faster than FLAIR and uses 27 times less memory (Additional file 2: Table S6).

## Discussion

The new StringTie2 system focuses on extending StringTie1’s capacity to handle long-read data, mostly by cleaning noise from the read alignments and by implementing more efficient data structures (see the “Methods” section). Our results show that this new re-engineering of StringTie also improved its assembly accuracy on short-read RNA-seq data. On both real and simulated data sets, StringTie2 is more accurate than Scallop. On all short-read data sets evaluated here, StringTie2 obtained better sensitivity, higher quantification accuracy, faster run time, and lower memory usage than Scallop. StringTie2 also had higher precision on all but one sample (ERR986114, Fig. 2), where it obtained slightly lower precision although it assembled many more correct transcripts than Scallop. We found that the drop in precision was likely due to transcripts being split by drops in read coverage, which can create two incorrect transcripts. An examination of intron-level accuracy revealed that StringTie2 was more accurate than Scallop on all data sets at that level (Additional file 2: Table S2). We also found that adjusting the parameters for both StringTie2 and Scallop for *Z. mays* improved the performance of both systems on that data, demonstrating that default parameters may not be optimal for all datasets. A systematic process for tailoring parameters to a particular dataset was recently explored for both Scallop and StringTie [35].

Its ability to use super-reads introduces partial de novo assembly into StringTie2, which provides modest improvements in sensitivity, precision, and abundance estimation on real and simulated data.

The high error rates of long reads generated by third-generation sequencers present distinct challenges that make identifying the exact exon-structure of a transcript difficult. Alignments of high-error long reads generated from the same locus usually disagree with one another, particularly surrounding splice sites (Additional file 1: Figure S2 and Figure S3). They also often disagree about the presence or absence of particular exons, especially if the exons are small (Additional file 1: Figure S3 and Figure S4). The results shown here demonstrate that StringTie2 is the most accurate method for assembly of transcripts from long, high-error rate reads. This has the potential to greatly improve the sensitivity of analyses using long-read RNA-seq data, which in the past has relied primarily on reads that span transcripts end-to-end. The built-in consensus calling in StringTie2 should also lessen the need for a separate error correction step from tools such as TranscriptClean [29]. In addition to its fast runtime and small memory footprint, StringTie2 requires no dependencies and can be easily run as a single command, unlike tools such as FLAIR which consist of a series of scripts that may each rely on other tools. StringTie2 is also multi-threaded, which allows it to be run in parallel on multi-processor computers and can significantly reduce the “wall clock” runtime of assembly.

It is worth noting that StringTie2 and FLAIR had higher accuracy on the ONT direct RNA dataset than on the cDNA dataset, despite the fact that both datasets were sequenced from the same sample. Direct RNA sequencing currently produces lower yields than cDNA sequencing; however, both datasets combined reads from multiple flowcells resulting in high coverage for each. The decreased accuracy is likely due to slightly lower read accuracy for the cDNA (85%) versus RNA (86%) data and to the smaller average fragment lengths in the cDNA dataset [36]. We also observed that 22% of the transcripts assembled by StringTie2 from the cDNA data were fully contained within introns, versus only 4% of the assemblies from the direct RNA data, suggesting the presence of a greater number of incompletely processed transcripts in the cDNA.

Further development of long-read RNA-seq technologies will increase the usefulness of StringTie2. In the case of ONT reads, improvements to basecalling will improve alignment quality, which will further improve StringTie2’s assemblies. As third-generation sequencers increase their throughput, researchers will also be able to use long-read RNA-seq for accurate transcript-level quantification, which currently requires the higher throughput of short read (i.e., Illumina) sequencers. ONT direct RNA sequencing has additional unique capabilities which are only beginning to be explored, such as the ability to identify RNA base modifications and secondary structure from the raw signal [36]. Better transcriptome assemblies will aid these efforts because these read-level features can then be associated with the full transcripts.

## Conclusions

We have demonstrated that StringTie2 can assemble RNA-seq data into full-length transcripts using both high-accuracy short reads and high-error long reads. StringTie2 outperforms all comparable tools in both transcriptome quality and computational performance. The ability to assemble noisy long reads enables greater sensitivity in downstream analyses and will become increasingly useful as long-read RNA sequencing technologies mature.

## Methods

### Reference genomes and annotations

All human RNA-seq reads were mapped to the main chromosomes of GRCh38, not including the “alternate” and “random” scaffolds. The annotation used to compute the accuracy of transcriptome assemblies and to create the human short-read simulated data and the annotation-guided assemblies contains all full-length protein and long non-coding RNA transcripts from RefSeq, release GRCh38.p8. The *A. thaliana* RNA-seq reads were aligned to the TAIR10 assembly, and the full corresponding annotation was used for determining accuracy [37]. The *Z. mays* reads were aligned to the B73 RefGen assembly, and the full corresponding annotation was obtained from MaizeGDB [38].

### Simulated data

A short-read RNA-seq dataset containing 150 million 75-bp paired-end reads was generated using Flux Simulator [39] with all protein-coding and lncRNA transcripts on the main chromosomes of GRCh38. The parameters for the simulation were the ones recommended for *Homo sapiens* in Additional file 2: Table S4 from [39]. Long read simulated data for *S. cerevisiae* S288 (baker’s yeast), *D. melanogaster* r6 (fruit fly), and GRCh38.p7 was obtained from [33]. The long reads were simulated using either PacBio (one dataset for yeast, fruit fly, and human each) or MinION ONT profiles (one data set for fruit fly and one for human).

### Alignment and assembly parameters

All short-read datasets were aligned using HISAT2 [40] with default parameters. The PacBio and ONT datasets were aligned with minimap2 [41] (version 2.12) using the “-splice” option, which enables spliced alignment of noisy long reads. Super-reads were aligned using GMAP [42] because their error profile more closely resembles that of EST sequences, which aligners like minimap2 are not designed for.

All assemblies in Additional file 2: Table S1 were run using default parameters for both StringTie2 (version 2.0.0) and Scallop (version 0.10.2). The *Z. mays* samples in Additional file 2: Table S3 were run with the “-t -g 200” options in StringTie2 and the “--min_bundle_gap 200” option in Scallop. This increases the maximum allowable gap between reads within a transcript from the default 50 to 200 bp for both tools and disables trimming of transcripts when the read coverage drops below a given threshold at the 5′ or 3′ ends for StringTie2 (no corresponding option exists for Scallop). StringTie2 was run using the “-L” parameter for all long-read datasets. Three FLAIR sub-commands were run in sequence to obtain the GTF of covered transcripts: “align,” “correct,” and “collapse,” using the human reference genome and annotation described above where required.

### Accuracy metrics

Similarly to previous studies (e.g., [14]), we used the following metrics to report the accuracy of the transcriptome assemblies:

$$ \mathrm{Sensitivity}=\mathrm{TP}/\left(\mathrm{TP}+\mathrm{FN}\right) $$

$$ \mathrm{Precision}=\mathrm{TP}/\left(\mathrm{TP}+\mathrm{FP}\right) $$

where TP (or true positives) are correctly assembled transcripts, FP (or false positives) are transcripts that are assembled but do not match the reference annotation, and FN (or false negatives) are transcripts in the reference annotation that are missing from the assembly. Note that FP is not a true measure of false positives for real (as opposed to simulated) data, because the reference annotation is incomplete for essentially all eukaryotic genomes today. Thus, transcripts that do not match the annotation might nonetheless be correct, and a more accurate term might be “additional transcript predictions.” However, for the purposes of comparison between methods, and for consistency with previous studies, we retain this definition. Sensitivity and precision were determined by running gffcompare [43]. At the transcript level, an assembled transcript was considered correct if, in comparison to an annotated transcript, it shared all splice site boundaries exactly and the terminal exons ended within 100 bp of each other. Intron-level accuracy, shown in Additional file 2: Table S2, was calculated similarly, where an intron was considered correct if both ends precisely matched an annotated intron.

Relative percent change in sensitivity (*S*_*r*_) and precision (*P*_*r*_) of StringTie2 versus another method was computed as ${S}_r=100\times \frac{S_{1-}{S}_2\ }{S_2\ }$, and ${P}_r=100\times \frac{P_{1-}{P}_2\ }{P_2\ }$, where *S*_1_ and *P*_1_ are the sensitivity and precision of StringTie2, and *S*_2_ and *P*_2_ are the sensitivity and precision of the method which we compare it to (e.g., Scallop or FLAIR). For example, a 10% absolute increase in sensitivity from *S*_2_ = 20% to *S*_1_ = 30% would be reported as a relative increase of 50%.

For real datasets, we have no way to determine exactly what transcripts were truly present in the sample. Therefore, for the purpose of comparison, we defined the set of reference or “true” transcripts to be the union of all annotated transcripts correctly predicted by each tool on a given sample. This metric will overestimate the absolute sensitivity if there are transcripts that no tool predicts, but the relative sensitivity comparison will be accurate because the denominator is the same between samples and therefore cancels out.

### New data structures in StringTie2 compared to StringTie1

StringTie2 builds on our previously developed StringTie1 system, which introduced several key innovations, notably (1) a novel network flow algorithm to reconstruct transcripts and quantitate them simultaneously and (2) an assembly method to merge read pairs into full fragments in the initial phase [9]. StringTie2 maintains the same general framework for the assembly and quantification of transcripts but implements much more efficient data structures that overall lead to faster run times and much lower memory usage (see the “Results” section). It includes additional techniques designed to handle very long reads, including high error-rate reads produced by the third-generation sequencers, as well as the longer reads that result from the pre-assembly of short reads.

There are three main differences in the way StringTie2 stores aligned reads compared to StringTie1. The first difference is that instead of storing every read individually, StringTie2 collapses reads aligned to the identical location on the genome and keeps a count of how many alignments were collapsed. This simple change has a big impact on the memory required to store input data, because very highly expressed transcripts can sometimes reach a coverage of hundreds of thousands of reads per base. (see for instance Additional file 1: Figure S5, which illustrates the very high level of expression for the COL1A1 gene in sample SRR534291 that was collected from fetal lung fibroblasts.) However, implementing this change was quite complex, because it also required us to create a different method for storing the pairings between reads, as reads aligned at the same place do not necessarily have their “mates” (the second read in each pair) sharing the same alignments. Previously, for each read, StringTie1 stored a pointer to its pair. StringTie2 must instead store an array of pointers to all paired read alignments that are present in the data.

StringTie2 also differs from StringTie1 in its more aggressive strategy for identifying and removing spurious spliced alignments. If a spliced read is aligned with more than 1% mismatches, keeping in mind that Illumina sequencers have an error rate < 0.5%, then StringTie2 requires 25% more reads than usual (the default is 1 read per bp) to support that particular spliced alignment. In addition, if a spliced read spans a very long intron (more than 100,000 bp), StringTie2 accepts that alignment (and the intron) only if a larger anchor of 25 bp (10 bp is the default) is present on both sides of the splice site. Here the term “anchor” refers to the portion of the read aligned within the exon beginning at the exon-intron boundary.

Another improvement in StringTie2 is in its internal representation of its splice graph and of the reads aligned to that graph. Both the assembly of reads into transcripts, as well as the quantification of the resulting transcripts, require determining the compatibility between the reads (or fragments) and a path in the splice graph [9], which requires many searches of the overlaps between reads and the splice graph. In order to maximize the efficiency of such searches, StringTie1 uses a bit-vector representation of the splice graph, where the first *n* bits (*0* to *n-1*) correspond to all nodes in the splice graph, and bit n*i + j corresponds to a possible edge between nodes *i* and *j* in the splice graph, where *n* is the number of nodes in the graph and *i < j* (Additional file 1: Figure S6). Thus, there are n*(n-1)/2 bits for the possible edges. A read or a paired read will therefore be represented by a vector of bits where only the bits that represent the nodes or edges spanned by the read and its pair are set to 1. Because in general many of the nodes in the splice graph are not connected by edges, most bits in this bit-vector representation will be 0; therefore, StringTie2 replaces it with a sparse bit-vector data structure, where the bits can only correspond to a node or an edge appearing in the splice graph. Building more efficient data structures in StringTie2 greatly reduced the memory footprint of the StringTie system. On the three datasets from this study that were also examined in the original StringTie release, memory usage was reduced on average by a factor of 40 (Additional file 1: Figure S7).

### Assembly of long RNA-seq reads

Third-generation sequencing technologies (i.e., from PacBio and Oxford Nanopore sequencing instruments) have an error profile that consists mostly of insertion and deletions, as opposed to second-generation errors that are mostly substitutions. Insertion and deletions are harder to correct than substitutions, and the accuracy of methods for correcting them is generally low [44]. Further complicating matters, aligning long reads correctly around splice sites is challenging, and mis-alignments lead to spurious edges in the splice graph, which in turn leads to incorrect transcript predictions [23].

To handle the high error rates in the long reads, we implemented two new techniques in StringTie2. First, we correct potentially wrong splice sites by checking all the splice sites present in the alignment of a read with a high-error alignment rate. If a splice site is not supported by any low-error alignment reads, then we try to find a nearby splice site (within 10 bp, by default) that is supported by the most alignments among all nearby splice sites. If we can find such a splice site, then we adjust the read alignment to match it. While this technique greatly reduces the false alignments around the splice sites, it does not eliminate the presence of spurious false exons introduced by random sequencing insertion errors. Pruning edges that are not supported by a minimum number of spliced reads, as described above, eliminates some of the false-positive edges. However, in regions of very high within-transcript sequence coverage, there may still be too many spurious nodes and edges in the splicing graph, which in turn may cause StringTie1 to hang indefinitely. To improve StringTie2’s efficiency in such cases, we designed and implemented a pruning algorithm that reduces the size of the splicing graph to a more realistic size (see Additional file 1: Algorithm S1). This algorithm removes edges in the graph starting from the edge least supported by reads to the most supported edge, until the number of nodes in the splicing graph falls under a given threshold (by default 1000 nodes). Pruning edges in the splicing graph will also change the internal representation of the long reads affected by the pruning. For instance, a long read that spans a node that is no longer part of the splicing graph might be represented as an interrupted read instead of a one continuous read, similar to how two paired reads are represented (see Additional file 1: Figure S6b).

### Super-read construction and quantification

Super-reads were constructed using code adapted from the MaSuRCA assembler [19]. MaSuRCA builds a *k*-mer lookup table out of every sequence of length *k* (*k*-mers) in the input reads. It uses this to create “*k*-unitigs,” which are defined as sequences of maximal lengths such that every *k*-mer except the first and last has a unique preceding and following *k*-mer. Super-reads are then constructed by matching each *k*-mer at the ends of each short read to a unique *k*-unitig, effectively extending the short read as far as there is a unique extension. Note that it is possible for a super-read to contain multiple short reads, and for a short read to be contained in multiple super-reads. Not all short reads are assigned to a super-read, so both super-reads and unassigned short reads are used for assembly.

Prior to the construction of the *k*-mer lookup table, MaSuRCA uses QuorUM [45] to correct errors in the short reads. The built-in parameters that it uses for genome assembly are not optimal for transcriptome assembly. For example, these parameters include a minimum number of times a *k*-mer must appear to be considered high quality, which is appropriate for genome assembly where all sequences should be covered uniformly, but not for transcriptome assembly, where some transcripts may have coverage as low as a single read. Therefore, we modified these routines to remove the minimum *k*-mer count thresholds used for error correction. There are also certain cases where the first and/or last *k-1* bases of a super-read can extend into alternatively spliced exons, which could mislead the assembly process. To alleviate this problem, StringTie2 ignores the first and last *k-1* bases of aligned super-reads.

Because many reads may be collapsed into a single super-read, StringTie2 needs a coverage estimate with every super-read in order to calculate the expression level of any transcript with super-reads aligned to it. To estimate coverage, we first find every super-read containing each short read by matching the *k*-unitigs. A read is assigned to a super-read if its *k*-unitigs are contained in the super-read in the same continuous order (or reverse order for the opposite strand), which happens if and only if the read (or its reverse complement) is an exact substring of the super-read. During this step, we only consider super-reads that have been aligned to the reference genome. After read assignment, we use an expectation-maximization algorithm to estimate coverage for each super-read. The initial estimate sums the coverage of each read or fragment uniquely assigned to one super-read. Each iteration then recomputes coverage for every super-read by distributing coverage from each read proportionally to the previous super-read coverage estimate. This is analogous to how StringTie2 distributes coverage between transcripts. We report the computed coverage for each super-read using a special tag in the SAM output file, which is then merged with an aligned short-read SAM file for input to StringTie2, which uses the super-reads to weight the paths that they match in the splice graph.

## Supplementary information

**Additional file 1: Algorithm S1.** Splicing graph pruning algorithm. **Figure S1.** Sensitivity and precision of StringTie2 versus StringTie1 on simulated data. **Figure S2.** An IGV snapshot of aligned ONT direct RNA reads in the region of gene C1orf174. **Figure S3.** An IGV snapshot of aligned simulated PacBio RNA reads from transcript uc060qvn.1. **Figure S4.** An IGV snapshot of aligned simulated PacBio RNA reads from transcript uc284pkq.1. **Figure S5.** An IGV snapshot of a extremely highly expressed transcript in the COL1A1 gene. **Figure S6.** a) Bit vector representations of a splice graph, an assembled transcript, and a fragment with two paired reads sequenced. b) Example of a long read aligning to the splice graph. **Figure S7**. Memory usage for StringTie1 and StringTie2.

**Additional file 2: Table S1.** Short-read accuracy. **Table S2.** Increased gap corn. **Table S3.** Short-read profiling. **Table S4.** Long-read accuracy. **Table S5**. Long-read profiling.

**Additional file 3.** Review history

## Data Availability

StringTie2 is implemented in C++ and is freely available as open-source software under the MIT license at https://github.com/gpertea/stringtie [46]. Ten of the short-read RNA-seq datasets used in this study were also used by Shao and Kingsford in their evaluation of Scallop [11] and can be found on the Sequence Read Archive under the following accessions: SRR307903, SRR315323, SRR315334, SRR534307, SRR545723, SRR307911, SRR387661, SRR534291, SRR534319, and SRR545695. Three of these ten—SRR534291, SRR534319, and SRR545695—were used in the original StringTie study [9], which described StringTie1. We also examined 13 short-read samples randomly selected from the GEUVADIS dataset [47] that can be found on the European Nucleotide Archive under the following accessions: ERR188021, ERR188022, ERR188041, ERR188060, ERR188063, ERR188073, ERR188107, ERR188311, ERR188341, ERR188462, ERR204883, ERR204926, and ERR204965. The five *Arabidopsis thaliana* datasets were obtained from [48] and can be found on the European Nucleotide Archive: ERR1886195, ERR1886317, ERR1886341, ERR1886384, and ERR1886412. The five *Z. mays* datasets were obtained from [49] and can be found on the European Nucleotide Archive: ERR986085, ERR986103, ERR986106, ERR986108, and ERR986114. The “full length” and “not full length” PacBio datasets were downloaded from http://datasets.pacb.com.s3.amazonaws.com/2014/Iso-seq_Human_Tissues/list.html. The ONT direct RNA-seq and cDNA datasets are from the NA12878 RNA sequencing consortium [36].
