# Supplementary material for: Transcriptome assembly from long-read RNA-seq alignments with StringTie2
Source: Genome Biol. 2019 Dec 16;20:278. doi: 10.1186/s13059-019-1910-1 (PMC6912988; doi:10.1186/s13059-019-1910-1)
Supplement: Supplementary file 1 — Additional file 1: Algorithm S1. Splicing graph pruning algorithm. Figure S1. Sensitivity and precision of StringTie2 versus StringTie1 on simulated data. Figure S2. An IGV snapshot of aligned ONT direct RNA reads in the region of gene C1orf174. Figure S3. An IGV snapshot of aligned simulated PacBio RNA reads from transcript uc060qvn.1. Figure S4. An IGV snapshot of aligned simulated PacBio RNA reads from transcript uc284pkq.1. Figure S5. An IGV snapshot of a extremely highly expressed transcript in the COL1A1 gene. Figure S6. a) Bit vector representations of a splice graph, an assembled transcript, and a fragment with two paired reads sequenced. b) Example of a long read aligning to the splice graph. Figure S7. Memory usage for StringTie1 and StringTie2. [file 13059_2019_1910_MOESM1_ESM.docx]

Supplemental Materials

**Algorithm S1. *Splicing graph pruning algorithm***

**Input**.

- splicing graph *G=(V,E)* where *V* and *E* represent the set of nodes and edges in the graph, respectively, and *n_i_* comes before *n_i+1_* on the genomic sequence *for* $\forall i\leq\left| V \right|-1$*, V={n_1_,n_2_,…,n_|V|_}*
- an upper threshold *m* on the number of nodes
- $W={\{w_{e}\}}_{e\in E}$ a set of coverages of all edges in *E*.

**Output**. Pruned graph *G^p^=(V^p^,E^p^)* with *|V^p^| ≤ m*

**Algorithm**.

1. Sort edges in *G* such that if *E={e_1_,e_2_,…,e_|E|_}* then $w_{e_{i+1}}\geq w_{e_{i}} \forall i\leq\left| E \right|-1$
2. *i=0, V^p^=V, E^p^=E*
3. *while | V^p^|* $>$ m *do*
   1. remove edge *e_i_* from *E^p^*
   2. *for* *n={n_s_, n_e_ }* where edge *e_i_* links nodes *n_s_* and *n_e_* do

*if n* has no edges connecting it to other nodes in *G^p^*

remove node *n* from *V*; *|V^p^|--*

*else if* *n_s_* and *n_s+1_* are adjacent on the genomic sequence (there is no intron to separate them) and there are no edges linking *n_s_* and *n_j_*, *s+1<j*, *then*

merge nodes *n_s_* and *n_s+1_*; *|V^p^|--*

*if* *n_e-1_* and *n_e_* are adjacent on the genomic sequence (there is no intron to separate them) and there are no edges linking *n_j_* and *n_e_*, *j<e-1*, *then*

merge nodes *n_e-1_* and *n_e_*; *|V^p^|--*

*i++*

*end while*

Supplementary Fig. S1. Sensitivity and precision of StringTie2 versus StringTie (version 1.0.3) on simulated data. Sensitivity for both tools was controlled by varying the minimum abundance allowed for any predicted transcript, as a percentage of the most abundant transcript assembled at a given locus (parameter -f). Each dot on the plot corresponds to a run of StringTie (in blue) or StringTie2 (in green) with the parameter -f $\boldsymbol{i}$, where $\boldsymbol{i\in\{0.01,0.02,0.03,0.04,0.05,0.06,0.07,0.08,0.09,0.1\}}$.


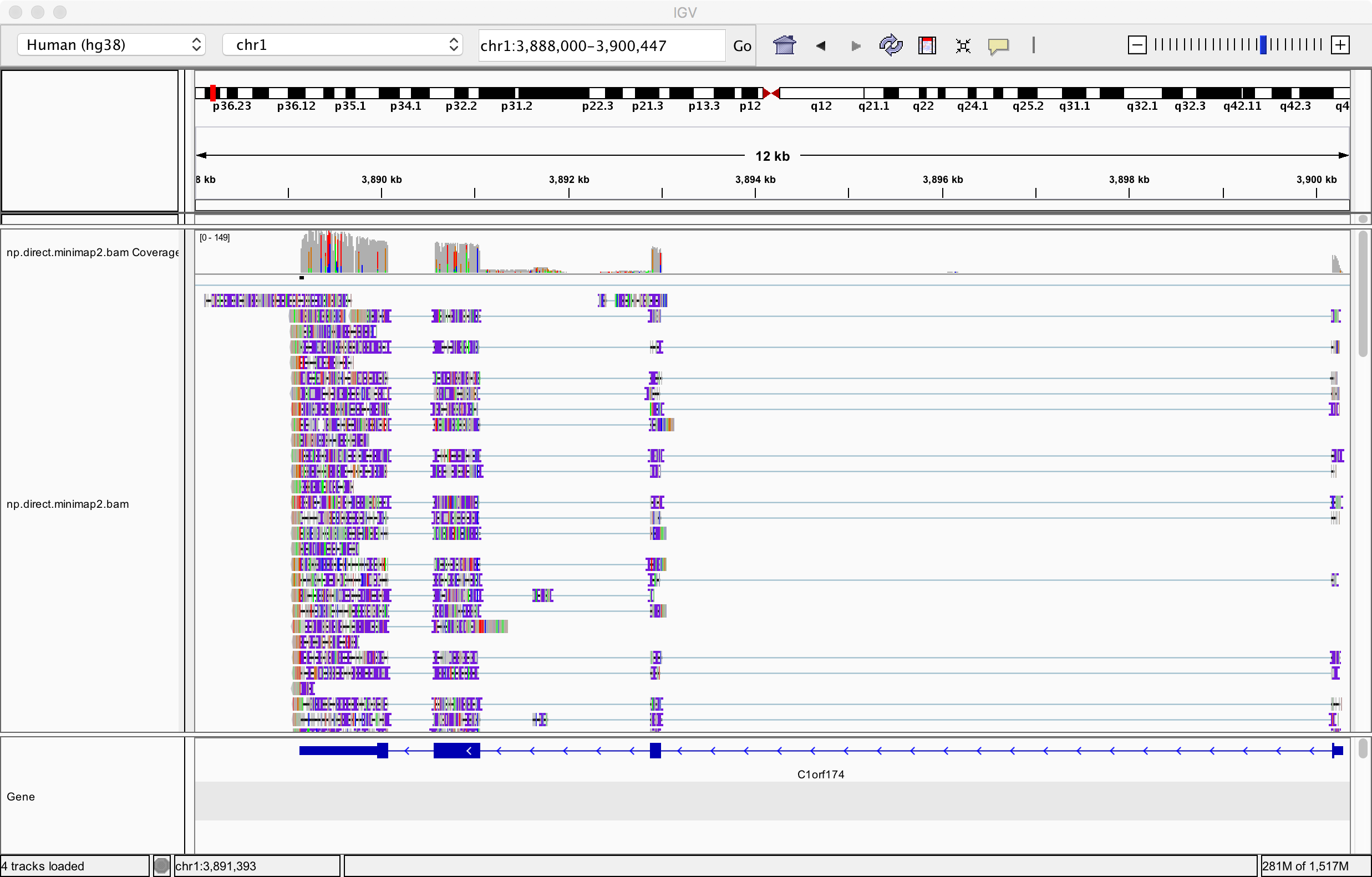


Supplementary Fig. S2. An IGV snapshot of aligned ONT direct RNA reads in the region of gene C1orf174. The alignments show many disagreements around the splice sites from C1orf174.


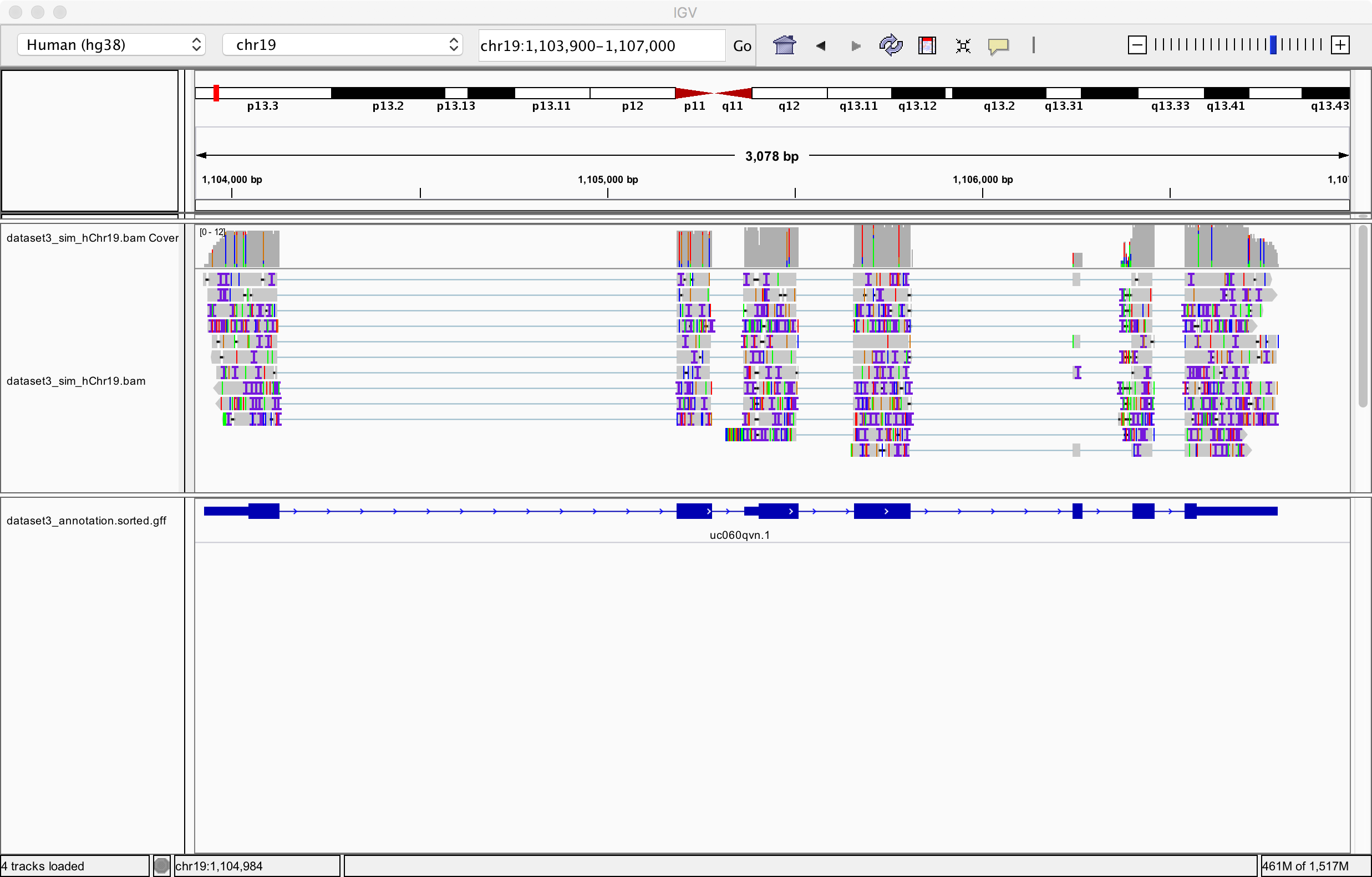


Supplementary Fig. S3. An IGV snapshot of aligned simulated PacBio RNA reads. All reads are simulated from transcript uc060qvn.1, pictured at bottom. Some of the alignments skip the 5^th^ exon in the transcript, and most alignments disagree around the splice sites.


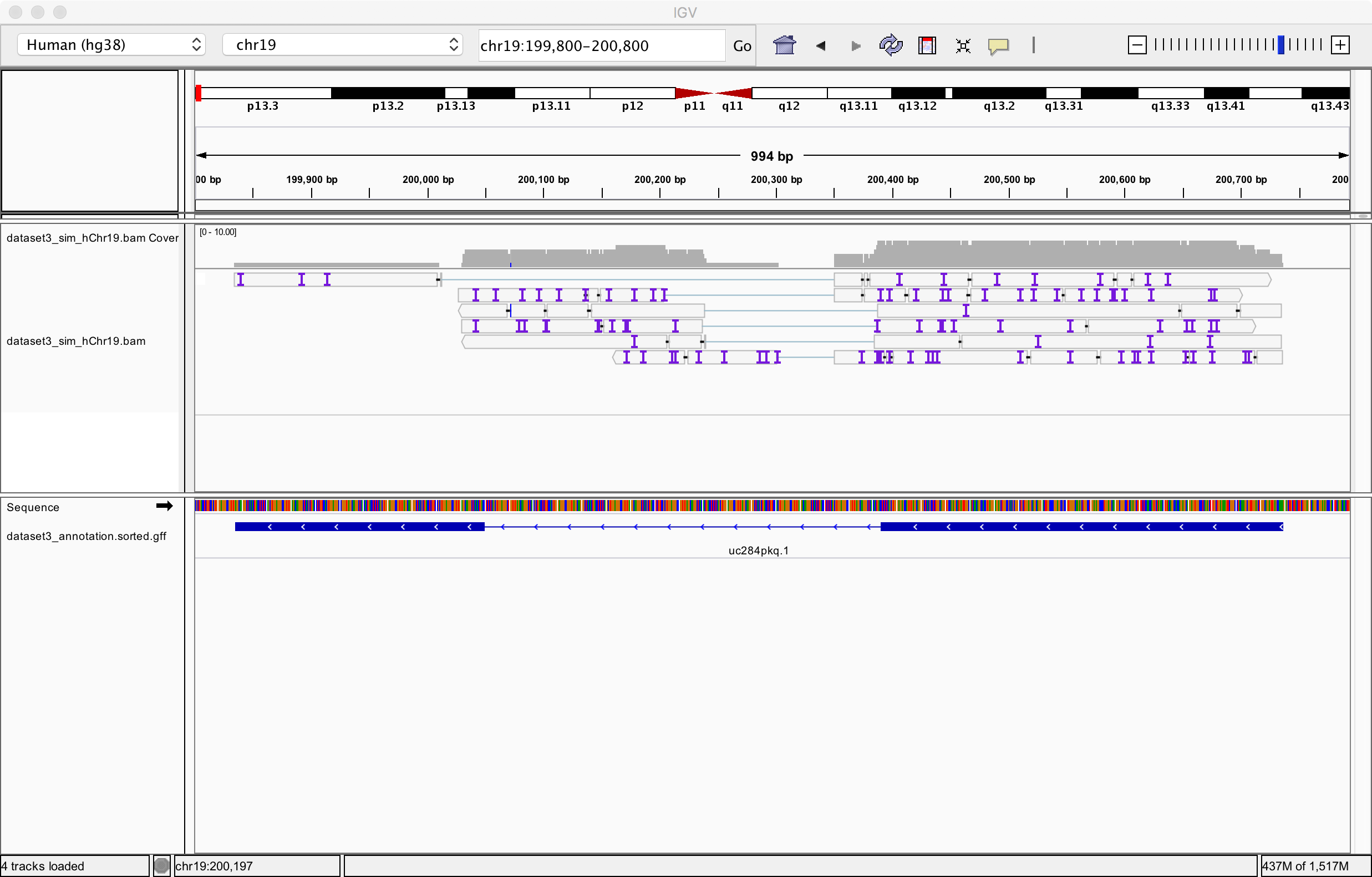


Supplementary Fig. S4. An IGV snapshot of aligned simulated PacBio RNA reads. All reads are simulated from transcript uc284pkq.1, pictured along the bottom, and yet none of the alignments correspond to the exact exon-intron structure of the transcript.


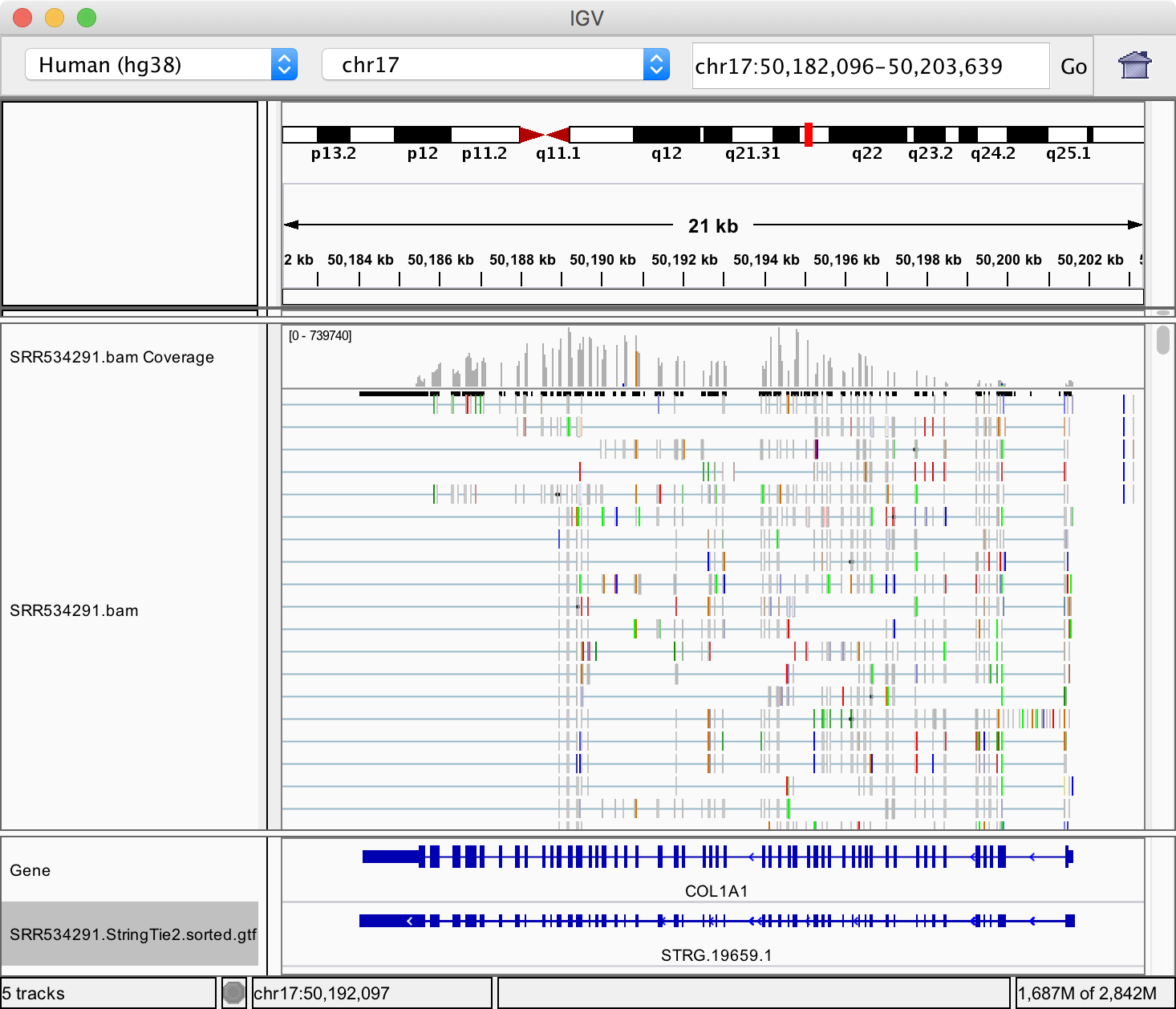


Supplementary Fig. S5. An IGV snapshot of a extremely highly expressed transcript in the COL1A1 gene captured by RNA-seq sample SRR534291, from the cytosol of fetal lung fibroblasts (GEO accession GSM981244). The coverage plot, shown as "SRR534291.bam coverage," peaks at a coverage above 739,000.

Supplementary Fig. S6. a) Representations of a splice graph (pictured at top), an assembled transcript T from the splice graph (pictured in blue as a path in the splice graph and as an exon-intron structure below the splice graph), a fragment F with two paired reads sequenced (pictured in black), and a bit vector corresponding to the splice graph with cells highlighted in blue representing the path of the transcript T, and cells set to 1 representing the fragment F. Note that the first row in the bit vector corresponds to all the nodes in the splice graph, while the cells *(i,j)* in the matrix below correspond to edges that link nodes *i* and *j* in the splice graph. B). b) Example of a long read before (in light green), and after (in dark green) pruning the splice graph to remove edges (1,2) and (2,4). The read spans three nodes in the graph before pruning (1, 2, and 4), while after pruning the two edges the long read appears as a paired read instead of a continuous read and spans only two nodes (1 and 4). The bit vector representations below show the bits set before and after pruning.

Supplementary Fig. S7. Memory usage for StringTie1 and StringTie2 on the three datasets also used in the experiments describing the original release of StringTie.
