# Supplementary material for: Transcriptome assembly from long-read RNA-seq alignments with StringTie2
Source: Genome Biol. 2019 Dec 16;20:278. doi: 10.1186/s13059-019-1910-1 (PMC6912988; doi:10.1186/s13059-019-1910-1)
Supplement: Supplementary file 3 — Additional file 3. Review history [file 13059_2019_1910_MOESM3_ESM.docx]

Review History

**First round of review**

**Reviewer 1**

**Were you able to assess all statistics in the manuscript, including the appropriateness of statistical tests used?**

Yes, and I have assessed the statistics in my report.

**Comments to author:**

The ability to generate long RNA-Seq reads with technologies such as PacBio or Nanopore reshapes the way researchers are able to investigate future transcriptomes. Long-read data has, besides a higher error rate and lower yield in comparison to short-read Illumina-derived data, the potential to generate full-length transcripts that span multiple exons. However, current reference-based assembly tools are not able to handle long reads accurately, in particular when derived from the more error-prone Nanopore technology.

Here, the authors present a major update of their reference-based transcriptome reconstruction tool StringTie, mainly to support more erroneous long reads next to short reads. They compare the tool against a state-of-the-art reference short-read assembler (Scallop, previously outperforming StringTie for some data) and also against long-read transcriptome assembly tools (FLAIR, Traphlor) that are currently rarely available. They use reasonable (simulated and real) short-read and long-read data sets for the evaluation (including data sets from Scallop that were previously used to outperform StringTie1 and also a Nanopore direct RNA-Seq sample). The new tool, StringTie2, can work with both, short- and long-read data, to assemble transcript isoforms based on a reference genome mapping. Furthermore, StringTie2 can construct super-reads from accurate short-read data and does not necessarily need an annotation (as FLAIR). In particular, the authors could also show the much better performance of StringTie2 when only using a reduced annotation set to guide the assembly process.

In general, I think that such an extension of StringTie is of high interest for the transcriptomic community because long-read RNA-Seq is already changing the way researchers are investigating transcriptomes and this trend will continue in the future. Reliable and fast computational methods to reconstruct transcript isoforms based on a reference genome are of great use.

The manuscript is very well written and clear to follow. The technical details and novel implementation principals behind StringTie2 are clear and reasonable. The supplemental material is comprehensive and usable. I was also able to easily download and execute StringTie2 with some test data.

In summary, I recommend publication after some minor suggestions and improvements are kindly considered by the authors:

[1]

Although I think that your evaluation metrics are reasonable (sensitivity and precision as defined in the manuscript), why did you not consider the usage of transcriptome evaluation programs such as BUSCO or rnaQUAST (e.g. compare Tab. 4 here: https://academic.oup.com/gigascience/article/8/5/giz039/5488105/#135058848)? At least rnaQUAST would give you a profound overview of all your calculated assemblies for one data set including meaningful metrics such as the 95%-assembled isoforms rate. In addition, it is not entirely clear to me when you define an assembled transcript as a TP hit. What are your thresholds to define a transcript as correctly assembled (something like at least 80% alignment length vs. a known transcript)?

[2]@Figure 4

Is there any explanation why FLAIR performed thus worse on the simulated PacBio S. cerevisiae data set (A panel) and Traphlor performed so much better here in comparison to Traphlors' performance on all the other data sets? This result is not really clear to me, because the performance on all other simulated data sets shows another general behavior for FLAIR/Traphlor.

[3]@Figure 6, Nanopore data

It's interesting to see that StringTie2 (and also FLAIR) performed better on the direct RNA-Seq Nanopore data (NPdirect) regarding sensitivity/precision compared to the NPcDNA data. Do both libraries derive from the same sample so that one could assume the same transcript composition? For me, this result is counterintuitive because the NPdirect sample should have 1) a higher error rate and 2) a much lower yield than the NPcDNA. But maybe the NPdirect sample has a higher amount of (near) full-length transcripts? Can the authors please comment on that?

Related to this question, how do you think does the higher error rate of the Nanopore dRNA-Seq sample (NPDirect) impacts the assembly with StringTie2? In comparison to NPcDNA?

[4]@Figures

In general, I have the feeling that the main figures can be improved in terms of panel arrangements and font sizes (for example in comparison to Supplementary Figure S1). The fonts of labels/legends are sometimes really huge (e.g., legend Fig. 4). Also please check the namings of tools (such as Flair - FLAIR) in the figures.

[5]

Please check for missing tool citations in the methods (HISAT2, minimap2, …)

[6]@Code and software usability

It would be great to also see a Bioconda recipe for StringTie2 or a Docker image in the future. But that's just frosting. Besides this, I was able to clone, make, and execute StringTie2 on provided test data.

[7]

This is more an outlook question. Did you consider using both, short and long reads as input for your method? StringTie2-Hybrid? I believe that combining the strengths of both technologies (Illumina vs. PacBio/Nanopore) could result in even higher sensitivity/precision metrics and completely reconstructed transcripts. Of course, currently there are not that many hybrid RNA-Seq data sets available but this might change in the near future. I do not consider the authors to include their thoughts on this in the current manuscript, however, this might be worth a sentence in the final outlook part if they like.

[8]

Please check language of Fig. 6 caption first sentence: "Number of correctly assembled transcripts correctly ..."

[9]

"FLAIR and Traphlor, the only other systems that can process long-read RNA sequencing data."

There is another software available, called SQANTI:

https://bitbucket.org/ConesaLab/sqanti/src/master/

https://genome.cshlp.org/content/early/2018/02/09/gr.222976.117

that the authors might also want to consider/mention in the manuscript. As far as I know, SQANTI also performs some kind of reference-based classification of long RNA-Seq reads.

[10]

Caption Supplementary Figure S2: "An IGV snapshot of a aligned ..." remove the "a"

Caption Supplementary Figure S5: "... expressed captured..." please check the wording

**Reviewer 2**

**Were you able to assess all statistics in the manuscript, including the appropriateness of statistical tests used?**

Yes, and I have assessed the statistics in my report.

**Comments to author:**

Major comment

Page 6 lines 8-14 … and page 12 lines 56-58

Here the authors give definitions of sensitivity and precision, the ones central for this work.

These definitions are standard in the area of pattern recognition, however, in case of sequence assembly topological properties of the objects (assembled transcripts) make the two metrics asymmetrical. Particularly, a missing short bridge between two correctly predicted parts of a transcript makes two negative events for precision, while adding this bridge would immediately flip the coin to a single positive count for sensitivity. Topological considerations would suggest that errors made in assembly of a single true transcript could produce multiple false positives along with only single false negative.

I would expect that the authors would comment on these properties of sensitivity and precision. In fact, they have made observation of disproportional low precision, in comparison with sensitivity, in genomes with high repeat content (Suppl. Table).

Minor comments

Page 2 line 30

Reads of this length usually span no more than one intron …

More precise is to say -

… no more than one splice junction …

Page 8 line 27

Spelling of StringTie2

Page 11 lines 59-60

Should the names of the species be italicized?

Page 14 line 60

the total size of the bit vector was not defined … notation n*i+j is not immediately obvious

**Authors Response**

**Point-by-point responses to the reviewers’ comments:**

Responses to Reviewer #1:

[… introduction omitted…]

In summary, I recommend publication after some minor suggestions and improvements are kindly considered by the authors:

[1]

Although I think that your evaluation metrics are reasonable (sensitivity and precision as defined in the manuscript), why did you not consider the usage of transcriptome evaluation programs such as BUSCO or rnaQUAST? At least rnaQUAST would give you a profound overview of all your calculated assemblies for one data set including meaningful metrics such as the 95%-assembled isoforms rate. In addition, it is not entirely clear to me when you define an assembled transcript as a TP hit. What are your thresholds to define a transcript as correctly assembled (something like at least 80% alignment length vs. a known transcript)?

*Response: We have added a new supplemental table to provide additional evaluation metrics, as we explain below. First, in response to the reviewer’s question about BUSCO: we have used BUSCO and its predecessor, CEGMA, for quite a few of our whole-genome sequencing projects in the past. BUSCO's primary purpose is to estimate the completeness of a newly assembled genome, by determining how many single-copy genes (from a small set of curated genes) are present and intact. That doesn’t really fit our purpose here for several reasons; e.g., we don’t expect all BUSCO genes to be expressed in any particular sample, and the BUSCO genes are only a small subset of all genes. More important, though, is that we know exactly which transcripts were expressed in our simulated datasets, so we can do much better, by computing exactly which transcripts were correctly reconstructed. For the real datasets, we evaluated accuracy using a convention established in earlier papers (including the Scallop paper and our own previous paper on StringTie1): using the annotation available for the human genome (and Arabidopsis thaliana and Zea mays), we compare all assembled transcripts to those annotations. We judge a transcript to be correct if it precisely matches, at all intron boundaries, the annotated transcript. We can also identify partial matches and characterize these as well, which we expand upon in the new Supplementary Table S2 (see below). This allows us to evaluate our output (and the output of other programs) much more comprehensively than if we just used the BUSCO genes.*

*We also looked at rnaQUAST, but that package is designed to assess de novo transcriptome assembly, as opposed to the reference-guided assemblies that StringTie2 outputs. rnaQUAST requires predicted transcripts to be provided as a FASTA file, which it then aligns to the genome. StringTie2 outputs a GTF file, which includes the genome coordinates of all exons in each transcript, so there is no need for this alignment step, which could (and indeed is likely to) introduce errors.*

*However, the gffcompare program that we use for comparing GTF (or GFF) files provides many more statistics than we included in our original manuscript. To address the reviewer’s question, we have added Supplementary Table S2 which contains intron-level sensitivity and precision, to provide a finer-grained picture of the results than our transcript-level comparisons. We have added the following explanation, referring to this new Supplementary Table S2, in the Methods section (under Accuracy metrics) describing how we match predicted introns and transcripts to annotations:*

*At the transcript level, a transcript was considered correct if, in comparison to an annotated transcript, it shared all splice site boundaries exactly and the terminal exons ended within 100bp of each other. Intron-level accuracy, shown in Supplementary Table S2, was calculated similarly, where an intron was considered correct if both ends precisely matched an annotated intron.*

[2] @Figure 4

Is there any explanation why FLAIR performed thus worse on the simulated PacBio S. cerevisiae data set (A panel) and Traphlor performed so much better here in comparison to Traphlors' performance on all the other data sets? This result is not really clear to me, because the performance on all other simulated data sets shows another general behavior for FLAIR/Traphlor.

*Response: We cannot state with confidence why FLAIR or Traphlor perform the way they do (after all, these are not our programs), but we think the answer here is that Traphlor doesn’t handle multi-exon genes very well. Only 4.4% of the yeast transcripts contained introns, which makes that dataset very different from the human and Drosophila data sets. To cast light on this issue, without getting into speculation, we added the following text to the manuscript (p. 8, in the paragraph beginning “Traphlor had lower sensitivity and precision than StringTie2 and FLAIR…”):*

*Note that only 4.4% of the simulated transcripts in the S. cerevisiae PacBio dataset contain multiple exons, a much smaller proportion than the other datasets, which may explain why Traphlor performed relatively well on this data while performing poorly on the human and Drosophila data.*

*To test this hypothesis a bit further, we compared the performance of FLAIR and Traphlor on the single-exon transcripts (excluding all multi-exon transcripts) in the simulated huamn and D. melanogaster PacBio datasets, shown in the table below. We see that Traphlor has higher sensitivity than FLAIR on single-exon transcripts, but far lower sensitivity on spliced transcripts. This supports our hypothesis, although we still don’t know why FLAIR performed so badly on the yeast data.*

*Sensitivity (%)	Precision (%)*

*FLAIR	Traphlor	Diff	FLAIR	Traphlor	Diff*

*Drosophila spliced	71.2	5.1	-66.1	91.4	27.2	-64.2*

*Drosophila unspliced	47.9	92.1	44.2	92.7	68.1	-24.6*

*Human spliced	84.0	7.0	-77.0	91.7	17.5	-74.2*

*Human unspliced	51.6	86.7	35.1	48.3	23.2	-25.1*

*Because this question is somewhat off-topic, and because this table only gives suggestive evidence, we are not including the table in the paper, but we provide it here to answer the reviewer’s question.*

[3]@Figure 6, Nanopore data

It's interesting to see that StringTie2 (and also FLAIR) performed better on the direct RNA-Seq Nanopore data (NPdirect) regarding sensitivity/precision compared to the NPcDNA data. Do both libraries derive from the same sample so that one could assume the same transcript composition? For me, this result is counterintuitive because the NPdirect sample should have 1) a higher error rate and 2) a much lower yield than the NPcDNA. But maybe the NPdirect sample has a higher amount of (near) full-length transcripts? Can the authors please comment on that?

Related to this question, how do you think does the higher error rate of the Nanopore dRNA-Seq sample (NPDirect) impacts the assembly with StringTie2? In comparison to NPcDNA?

*Response: Actually, the direct RNA dataset does not appear to be lower quality than the cDNA dataset; the bioRxiv paper reporting these datasets (which we reference, and which was just published on Nov 19 in Nature Methods) says that the direct RNA has an accuracy of 86% and the cDNA has an accuracy of 85%. Interestingly, in the cDNA dataset we found a large number of reads that were fully contained in the intron of a transcript. This resulted in StringTie2 predicting that ~22% of the cDNA transcripts were entirely contained in introns, compared to ~4% on direct RNA.*

*We added a paragraph in the discussion (pp 10-11) to discuss this question, as follows:*

*It is worth noting that StringTie2 and FLAIR had higher accuracy on the ONT direct RNA dataset than on the cDNA dataset, despite the fact that both datasets were sequenced from the same sample. Direct RNA sequencing currently produces lower yield than cDNA sequencing; however both datasets combined reads from multiple flowcells resulting in high coverage for each. The decreased accuracy is likely due to slightly lower read accuracy for the cDNA (85%) versus RNA (86%) data and to the smaller average fragment lengths in the cDNA dataset [36]. We also observed that 22% of the transcripts assembled by StringTie2 from the cDNA data were fully contained within introns, versus only 4% of the assemblies from the direct RNA data, suggesting the presence of a greater number of incompletely processed transcripts in the cDNA.*

[4]@Figures

In general, I have the feeling that the main figures can be improved in terms of panel arrangements and font sizes (for example in comparison to Supplementary Figure S1). The fonts of labels/legends are sometimes really huge (e.g., legend Fig. 4). Also please check the namings of tools (such as Flair - FLAIR) in the figures.

*Response: Thanks for pointing this out. We fixed FLAIR to show it in all caps in the figure legends, adjusted the font sizes in the legend of Figure 4, and made Figure 1’s axis labels consistent.*

[5]

Please check for missing tool citations in the methods (HISAT2, minimap2, …)

*Response: Thanks for catching this. We added the citations to these tools in the Methods section, where they were missing before.*

[6]@Code and software usability

It would be great to also see a Bioconda recipe for StringTie2 or a Docker image in the future. But that's just frosting. Besides this, I was able to clone, make, and execute StringTie2 on provided test data.

*Response: This is something we would like to do in the future, but for now we are maintaining it only in github.*

[7]

This is more an outlook question. Did you consider using both short and long reads as input for your method? StringTie2-Hybrid? I believe that combining the strengths of both technologies (Illumina vs. PacBio/Nanopore) could result in even higher sensitivity/precision metrics and completely reconstructed transcripts. Of course, currently there are not that many hybrid RNA-Seq data sets available but this might change in the near future. I do not consider the authors to include their thoughts on this in the current manuscript, however, this might be worth a sentence in the final outlook part if they like.

*Response: This type of hybrid dataset would likely work well with StringTie2, as a means of more accurately assembling full-length transcripts, and in fact it sort of works now, but we want to test it more thoroughly before describing it in a publication. We don’t see anyone (yet) using long reads for quantification, due to their higher cost, and there would also be challenges using two sets of reads that don’t necessarily come from the same sample or conditions. We will definitely explore this in our future work, but we didn’t add a sentence about it because we don’t want users to think that we are recommending (yet) the use of both short and long reads in a single run.*

[8]

Please check language of Fig. 6 caption first sentence: "Number of correctly assembled transcripts correctly ..."

*Response: Thanks for catching this. It is now fixed.*

[9]

"FLAIR and Traphlor, the only other systems that can process long-read RNA sequencing data."

There is another software available, called SQANTI:

that the authors might also want to consider/mention in the manuscript. As far as I know, SQANTI also performs some kind of reference-based classification of long RNA-Seq reads.

*Response: The reviewer is correct, there are other tools that can process long-read RNA-seq, including SQANTI which we were aware of. However, we want to highlight that there are few tools that can process both PacBio and ONT data. We have changed that sentence to “FLAIR and Traphlor, the only other systems that can process both PacBio and ONT long-read RNA sequencing data.” SQANTI is a PacBio-specific tool, so we now cite it in in second-to-last paragraph of the introduction when discussing PacBio-specific tools, which all rely on PacBio IsoSeq specific features, such as the 3’ and 5’ adapters. The revised text (p. 5) reads:*

*Various tools have recently been developed to correct errors and/or extract full-length transcripts from genome alignments of long RNA-seq reads. Tools that process full-length transcripts in PacBio Iso-Seq reads, including ToFU [26], TAPIS [27], and SQANTI [28], cannot assemble reads that only partially cover transcripts into full-length transcripts, nor can they be applied to ONT reads due to their reliance on identifying 5’ and 3’ ends based on PacBio-specific adapters.*

[10]

Caption Supplementary Figure S2: "An IGV snapshot of a aligned ..." remove the "a"

Caption Supplementary Figure S5: "... expressed captured..." please check the wording

*Response: Thanks for catching these. Both are now fixed.*

Responses to Reviewer #2:

Major comment

Page 6 lines 8-14 … and page 12 lines 56-58

Here the authors give definitions of sensitivity and precision, the ones central for this work.

These definitions are standard in the area of pattern recognition, however, in case of sequence assembly topological properties of the objects (assembled transcripts) make the two metrics asymmetrical. Particularly, a missing short bridge between two correctly predicted parts of a transcript makes two negative events for precision, while adding this bridge would immediately flip the coin to a single positive count for sensitivity. Topological considerations would suggest that errors made in assembly of a single true transcript could produce multiple false positives along with only single false negative.

I would expect that the authors would comment on these properties of sensitivity and precision. In fact, they have made observation of disproportional low precision, in comparison with sensitivity, in genomes with high repeat content (Suppl. Table).

*Response: It is true that transcript-level sensitivity and precision are asymmetric, and that provide an incomplete assessment of the transcriptome quality. We used these statistics because they are relatively easy to compute and interpret, and because they are used pervasively in related previous work (e.g., Pertea et al. 2015, Shao and Kingsford 2017). The specific case that the reviewer brings up, a short bridge missing within a transcript, is quite relevant to the Z. mays dataset where Scallop was more precise than StringTie2. We realized that intron-level sensitivity and precision will help to complete the picture here, since most introns remain intact when a transcript is split. We have added a new set of intron-level statistics in Suppl Table S2, and clarified the nuances of these statistics in several places in the manuscript, as follows:*

*We revised the description of transcript-level sensitivity and precision on real data in the third paragraph of “Results: Transcriptome assembly of short RNA-seq reads,” which now reads:*

*For this data, we cannot know with certainty which transcripts were expressed in each dataset, nor can we know their precise expression levels. However, it is generally safe to assume that an assembler is more sensitive if it assembles more transcripts matching known annotations (i.e., transcripts from a published database of known genes), and that it is more precise if the known transcripts represent a higher proportion of all the transcripts that are output by the assembler. Therefore, for each sample we calculate sensitivity and precision by considering the number of assembled transcripts that match annotations as correct ones (i.e., true positives), and counting all additional transcripts as incorrect (also see Methods).*

*We discussed one of the Zea mays samples ERR986114 in the fourth paragraph of “Results: Transcriptome assembly of short RNA-seq reads,” which we have revised to read:*

*Close inspection of the read alignments from this sample in IGV [32] revealed that there were many gaps in coverage within individual transcripts, possibly due to the highly repetitive nature of the Z. mays genome. Examining finer-grained statistics revealed that the intron-level precision of StringTie2 was higher than Scallop on this dataset (Supplementary Table S2), suggesting that the reduced transcript-level precision could be due to split transcripts caused by drops in coverage, which would result in fewer full-length transcript matches while leaving most introns intact. In fact, adjusting corresponding parameters in StringTie2 and Scallop to allow for larger gaps in coverage (see Methods) resulted in higher sensitivity and precision for StringTie2 on all Zea mays datasets (Supplementary Table S3).*

*We also discussed sample ERR986114 in the first paragraph of the Discussion, which we have revised to read as follows:*

*StringTie2 also had higher precision on all but one sample (ERR986114, Fig. 2), where it obtained slightly lower precision although it assembled many more correct transcripts than Scallop. We found that the drop in precision was likely due to transcripts being split by drops in read coverage, which can create two incorrect transcripts. An examination of intron-level accuracy revealed that StringTie2 was more accurate than Scallop on all data sets at that level (Supplementary Table S2). We also found that adjusting the parameters for both StringTie2 and Scallop for Z. mays improved the performance of both systems on that data, demonstrating that default parameters may not be optimal for all datasets.*

Minor comments

Page 2 line 30

Reads of this length usually span no more than one intron …

More precise is to say -

… no more than one splice junction …

*Response: Actually, a read that spans an intron will cross 2 splice junctions, the donor and acceptor sites for that intron. We modified it to “…span no more than two exons, except in cases of very small exons.”*

Page 8 line 27

Spelling of StringTie2

*Response: This is now fixed.*

Page 11 lines 59-60

Should the names of the species be italicized?

*Response: Yes, thanks for catching. This is now fixed.*

Page 14 line 60

the total size of the bit vector was not defined … notation n*i+j is not immediately obvious

*Response: We clarified this further. The vector has one bit for each node in the splice graph and one additional bit for each edge. We use n as the number of nodes, so there are n(n-1)/2 edges, for a total of n(n+1)/2, as illustrated in Suppl Figure 6. However, most of the edges do not appear and are set to zero, allowing us to use a sparse bit-vector data structure (built into C++), which saves a considerable amount of memory. Suppl Figure 7 shows, on real data, how much less memory StringTie2 requires compared to StringTie1. The revised text now reads:*

*In order to maximize the efficiency of such searches, StringTie1 uses a bit-vector representation of the splice graph, where the first n bits (0 to n-1) correspond to all nodes in the splice graph, and bit n*i+j corresponds to a possible edge between nodes i and j in the splice graph, where n is the number of nodes in the graph and i<j (Supplementary Figure S6). Thus there are n*(n-1)/2 bits for the possible edges.*

*The text following this, which describes the sparse bit-vector data structure and refers to Suppl Figure 7, is unchanged.*

**Second round of review**

**Reviewer 1**

Thank you very much for the clear answers and the extra work. I am satisfied with the current state of the manuscript and recommend it for publication.
